# Precise Sequential DNA Ligation on A Solid Substrate: Solid-Based Rapid Sequential Ligation of Multiple DNA Molecules

**DOI:** 10.1093/dnares/dst032

**Published:** 2013-07-29

**Authors:** Eiji Takita, Katsunori Kohda, Hajime Tomatsu, Shigeru Hanano, Kanami Moriya, Tsutomu Hosouchi, Nozomu Sakurai, Hideyuki Suzuki, Atsuhiko Shinmyo, Daisuke Shibata

**Affiliations:** 1 Kazusa DNA Research Institute, 2-6-7 Kazusa-kamatari, Kisarazu, Chiba 292-0818, Japan; 2 Research Association for Biotechnology, Nishishinbashi Yasuda Union Bldg, 2-4-2 Nishi-shinbashi, Minato-ku, Tokyo 105-0003, Japan; 3 Graduate School of Biological Science, Nara Institute of Science and Technology, 8916-5 Takayama, Ikoma, Nara 630-0192, Japan; 4 Biotechnology Laboratory, Toyota Central Research and Development Laboratories, 41-1 Yokomichi, Nagakute, Aichi 480-1192, Japan

**Keywords:** DNA ligation, cloning, multiple DNA fragment assembly, functional genomics

## Abstract

Ligation, the joining of DNA fragments, is a fundamental procedure in molecular cloning and is indispensable to the production of genetically modified organisms that can be used for basic research, the applied biosciences, or both. Given that many genes cooperate in various pathways, incorporating multiple gene cassettes in tandem in a transgenic DNA construct for the purpose of genetic modification is often necessary when generating organisms that produce multiple foreign gene products. Here, we describe a novel method, designated PRESSO (precise sequential DNA ligation on a solid substrate), for the tandem ligation of multiple DNA fragments. We amplified donor DNA fragments with non-palindromic ends, and ligated the fragment to acceptor DNA fragments on solid beads. After the final donor DNA fragments, which included vector sequences, were joined to the construct that contained the array of fragments, the ligation product (the construct) was thereby released from the beads via digestion with a rare-cut meganuclease; the freed linear construct was circularized via an intra-molecular ligation. PRESSO allowed us to rapidly and efficiently join multiple genes in an optimized order and orientation. This method can overcome many technical challenges in functional genomics during the post-sequencing generation.

## 1. Introduction

Since the first description of recombinant DNA by Cohen *et al.* in 1973,^1^ scientists have dreamt of introducing novel characteristics into organisms for academic or industrial purposes, characteristics such as the production of desirable crops or consumable materials. Joining two or more DNA fragments via a ligase-catalyzed reaction (a ligation) is a fundamental technique in recombinant DNA technology and genetic engineering. The ligation of different DNA fragments can create new functional units. Many organisms can be genetically modified via recombinant DNA technology.

Recent advances in functional genomics have revealed that gene clusters are involved in certain biological pathways or metabolic processes.^2–9^ For instance, a defined series of enzymatic reactions are often required in individual metabolic pathways. Therefore, introduction of a heterologous metabolic pathway into a host species will often required that multiple genes are expressed in the host organisms. However, despite many advances in molecular biology and genetic engineering technologies, a rapid, simple, and efficient method for joining a series of many gene cassettes is lacking.

Stepwise cloning of restriction fragments is a commonly used method for connecting multiple DNA fragments. However, this method is time-consuming; moreover, it is often difficult to devise a cloning strategy because the number of unique restriction sites is often too limited. To address these difficulties and to construct long DNA fragments, several methods have been developed; MultiSite Gateway cloning, polymerase assembly multiplexing (PAM) method, In-Fusion system, and chemical syntheses of genomes are some of these methods. The MultiSite Gateway cloning is based on DNA recombination; a maximum of four DNA fragments can be assembled into one construct simultaneously, because the number of unique *att*B recombination site is limited. However, when more than five DNA fragments need to be joined, the Gateway methods also require many sequential reactions. The PAM method requires three steps for the construction of multi-cassette transgenes: polymerase cycling assemble of multiple oligonucleotides, overlap extension and annealing, and repair and ligation.^10^ The In-Fusion system is similar in principle to PAM; DNA fragments are amplified via PCR with oligonucleotide primers that carry extensions of >15 bp, and the In-Fusion exonuclease is used to chew from 3′ to 5′ at the fragment ends to produce single-stranded ends; the ends are annealed, and the DNA fragments are repaired and ligated in *Escherichia coli*.^11^ These methods for connecting multiple fragments proceed in a single step; therefore, the ligation reactions are faster than the stepwise cloning. But, these methods are often problematic when constructs with many gene cassettes are being built. It is difficult to ensure that the desired gene order and orientation and the correct nucleotide sequence are produced with any of these methods. Chemical syntheses of genome can be used to produce very large DNA constructs;^12,13^ however, this method often produces high error rates, and it is very expensive. Thus, the joining of multiple DNA fragments in a desired order is still labour intensive and time consuming.

**Figure 1.**
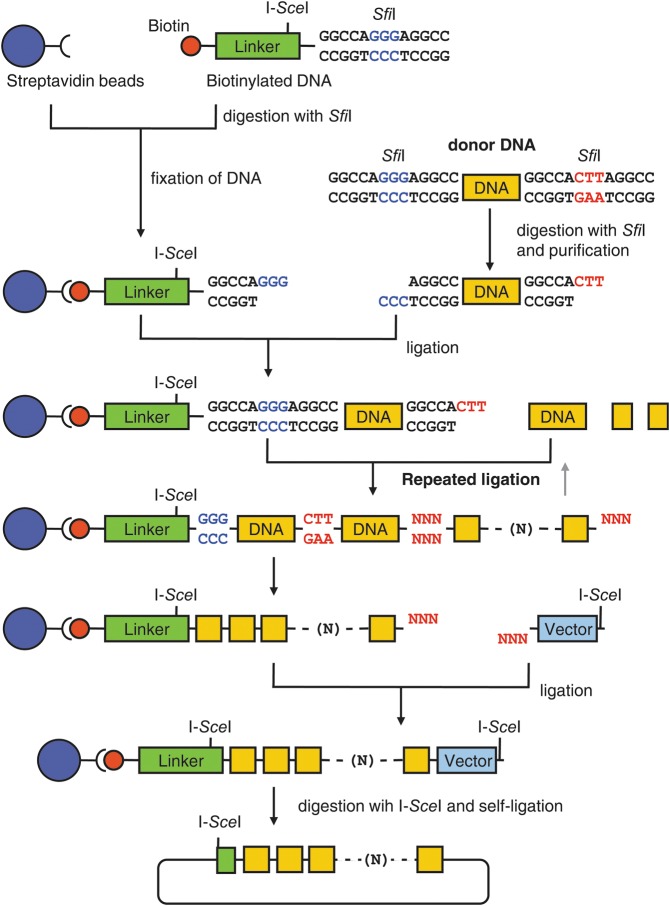
Schematic representation of the PRESSO method. The 5′-biotin-labelled DNA molecule with recognition sequences for a meganuclease, e.g. I-*Sce*I, and with a non-palindromic restriction site, e.g. an *Sfi*I site, is used as the initial acceptor molecule for a series of sequential ligations. The donor DNA molecules for the subsequent ligations are prepared via PCR; the 5′-primers are biotinylated, the 3′-primers are unmodified for the initial acceptor and biotinylated for the donor DNAs, and all primers except for the 5′-primer of the initial acceptor contain an *Sfi*I site. The amplified donor DNA fragments are digested with *Sfi*I and purified. For convenience, ∼200 bp DNA fragments are used as a spacer DNA (linker) in this study. The initial linker DNA molecules are attached to streptavidin-coated beads via the biotin label. The 3′-end of the initial acceptor DNA (the linker) is designed to be complementary with the 5′-end of the initial donor molecule, which has non-palindromic sites in 5′- and 3′-ends. The 3′-end of the first donor molecule is designed to be complimentary to the 5′-end of next donor DNA. Each ligation step is performed individually; a donor DNA is ligated to the growing end of a construct that is attached to a solid bead via the opposite biotin-labelled end; the beads are then washed and collected. In subsequent sequential reactions, the 5′-end of the next donor DNA molecule is ligated to the 3′-end of bead-bound DNA. In this manner, each donor DNA is connected to an acceptor DNA molecule that is attached to a solid bead. The final donor DNA molecules contain a vector sequence and a meganuclease, I-*Sce*I, recognition site. Therefore, the final products on the beads include an I-*Sce*I site on each end. The ligated DNA fragments are released from the bead via I-*Sce*I digestion; to construct a transformable plasmid, the newly freed linear fragments are circularized via an intra-molecular ligation. The circular plasmids are then used to transform *E. coli*; replicated plasmids can then be recovered from transformed cells.

Here, we describe an alternative method for the sequential ligation of multiple DNA fragments, the precise sequential DNA ligation on a solid substrate (PRESSO) (Fig. 1). This method is cost-effective and produces DNA constructs more accurately than do previously described methods.

## 2. Materials and methods

### 2.1. Materials

The name, sequence, and restriction site information for each primer used in this study are listed in Supplementary Table S1. In this study, a unique restriction enzyme site was added onto many of the primers, so that restriction digests could be used to confirm the final product (Fig. 3). All restriction enzymes (including *Sfi*I, *Bst*XI, and I-*Sce*I), KOD—Plus—Ver. 2 DNA polymerase, which exhibits high PCR fidelity, and *E. coli* DH5-α competent cells were purchased from TOYOBO (Osaka, Japan). We used Dynabeads® M-280 Streptavidin-linked beads (Invitrogen, Carlsbad, California, USA) and DNA ligation kit <Mighty mix> (TAKARA BIO, Otsu, Japan) as the solid phase for the sequential ligation reactions. In our preliminary experiments, we found that the efficiency of transformation of *E. coli* with the resultant plasmid carrying sequentially ligated DNA fragments varied significantly among reaction series that were examined by using some ligation mixture or ligation kits. The DNA ligation kit <Mighty mix> (TAKARA BIO) exhibited high efficiency enough to connect multiple DNA fragments using the PRESSO protocol. Usually 10–100 of *E. coli* colonies were obtained when we transformed highly efficient competent *E. coli* cells with the plasmid carrying a 20-kb DNA insert connected with 10 DNA fragments of 2 kb (Fig. 4). We recommend users to use the kit or to find a suitable one from available kits.

### 2.2. Synthesis of a spacer DNA

A biotin-conjugated DNA molecule with an I-*Sce*I recognition site was synthesized as the spacer molecule that was attached to the streptavidin-linked beads. First, two complementary oligonucleotides, I-*Sce*I-F and I-*Sce*I-R, which each contained a I-*Sce*I recognition sequence, were annealed to one another to generate a double-stranded DNA molecule; this molecule was then inserted between the *Eco*RI and *Pst*I sites of the multi-cloning region of pUC19. This plasmid, pUC19-I-*Sce*I, and two biotin-labelled primers, PUC-N-u (5′) and PUC-CS (3′), were to PCR amplify a biotin-labelled DNA fragment (242 bp). The amplified products were purified with the QIAquick PCR purification Kit (QIAGEN, Germantown, MD, USA). The purified samples were dissolved in 100 μl of digestion buffer, and digested at 50°C with 50 U of *Sfi*I overnight. The digestion products were purified with the QIAquick PCR Purification Kit (QIAGEN) and precipitated with ethanol.

### 2.3. Preparation of the initial donor DNA–streptavidin beads

Purified biotin-labelled linker DNA molecules (2 μg) were mixed with 30 μl of Dynabeads M-280 (Invitrogen) in binding solution [5 mM Tris–HCl (pH 7.5), 0.5 mM ethylenediaminetetraacetic acid (EDTA), and 1 M NaCl] for 2 h at room temperature; the beads were washed with the washing buffer [5 mM Tris–HCl (pH 7.5), 0.5 mM EDTA, and 1 M NaCl] according to the manufacture's protocol.

### 2.4. DNA fragments for the sequential ligation

**Figure 2.**
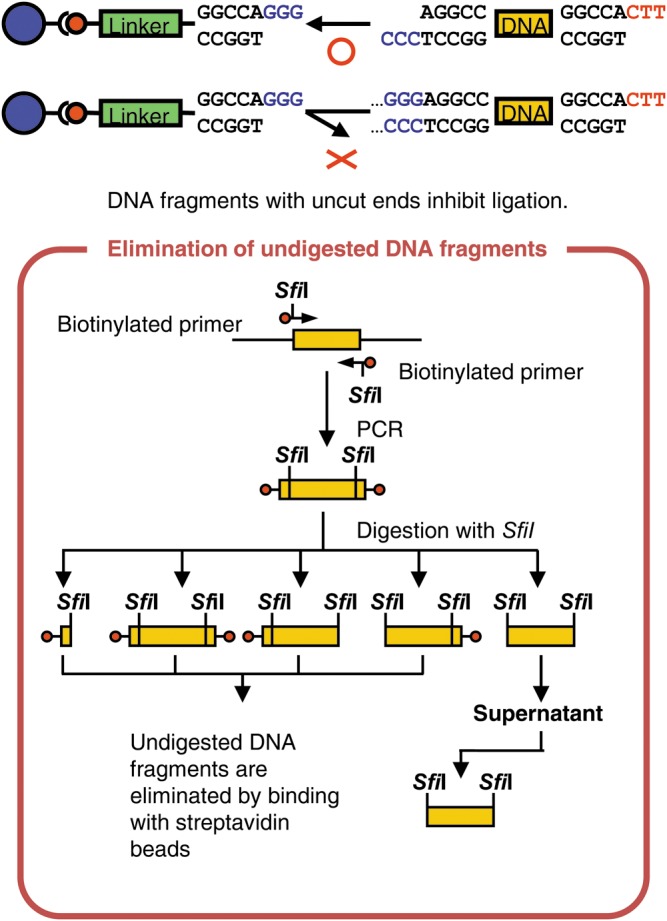
Purification of donor DNAs that were amplified with biotinylated primers. We purify donor DNA molecules to remove partially digested molecules. This step is often important to perform successful iterative ligations of multiple DNA fragments. Each donor molecule is amplified with biotinylated primers via PCR. The amplified fragments are digested with *Sfi*I. When the digested fragments are mixed with streptavidin beads, partially digested products should interact with the beads, but the fully digested molecules should not. The supernatants are collected, because they contain the fully digested products with the appropriate overhang sequences; these products are then used for the sequential ligations.

To demonstrate the utility of PRESSO, three sets of 10 DNA fragments were PCR amplified from *Arabidopsis thaliana* genomic clones (Supplementary Table S2) with biotin-labelled oligonucleotide primers that each contained an *Sfi*I restriction enzyme (Supplementary Table S1). The reactions were carried out for 30 cycles of 94°C for 30 s, 50°C for 30 s, and 72°C for 1 min. The amplification products were purified with the Wizard® SV Gel and PCR Clean-UP System (Promega, Madison, Wisconsin, USA), digested with *Sfi*I as described above, and then applied to streptavidin-linked magnetic beads to remove any undigested products (Fig. 2). *Sfi*I-digested DNA (2 μg in 300 μl) was mixed with streptavidin-linked beads (in 300 μl) for 1 h with gentle stirring. As undigested DNA fragments bound to the beads, the supernatant fractions were recovered to collect the fully digested products, which were then used for the sequential ligation reactions (see below).

### 2.5. Sequential ligation of DNA fragments

We dissolved 600 ng of the first donor DNA molecule in 30 μl of the ligation buffer from the DNA ligation kit (TAKARA BIO); this solution was then mixed with 30 μl of the mixture that contained streptavidin beads coated with biotin-labelled digested spacer DNA from pUC19-I-*Sce*I; 30 μl of ligase solution from the kit was then added. The mixture was incubated at room temperature for 1 h with gentle agitation on a rotator. The beads were then washed twice with Tris–EDTA (TE) buffer (200 μl for each wash). The beads were then sequentially incubated with each DNA fragment, in the appropriate order; each incubation was carried out in ligation buffer from the kit.

For the last ligation reaction, we prepared a modified pHSG299 (TAKARA BIO) vector, designated pHSG299_CSPS. The vector pHSG299 was digested with *Eco*RI and *Sph*I, and the following sequence was inserted to introduce four meganuclease recognition sites—PI-*Sce*I, PI-*Psp*I, I-*Sce*I, and I-*Ceu*I—into the vector; in this sequence (GAATTCgcggccgcggccggcccgggcTGCCATTTCATTACCTCTTTCTCCGCACCCGACATAGATtcttctACCCATAATACCCATAATAGCTGTTTGCCAtcttctTAGGGATAACAGGGTAATtcttctTCGCTACCTTAGGACCGTTATAGTTAggcgcgcctgcaggGCATGC), the upper case characters indicate, respectively, the following restriction enzyme recognition sites—*Eco*RI, PI-*Sce*I, PI-*Psp*I, I-*Sce*I, I-*Ceu*I, and *Sph*I. The modified vector pHSG299_CSPS was amplified with two primers, CSPS-N2-CAA-u and CSPS-C2-u (Supplementary Table S1), and digested with two enzymes, *Sfi*I and I-*Sce*I. The digestion product was used for the last sequential ligation.

### 2.6. Isolation of the ligation products

The beads that contained the complete PRESSO product were first washed with 1.5 ml of TE buffer, and then rinsed with 200 μl of I-*Sce*I buffer. The beads were then incubated with 50 U of I-*Sce*I at 37°C for 2 h in 50 μl of I-*Sce*I buffer. To release the DNA construct containing the array of fragments from the beads, the samples were heated at 60°C for 10 min with sodium dodecyl sulphate (final concentration, 0.1%), and the supernatant was collected. The DNA was precipitated with ethanol, and then dissolved in 200 μl of buffer that contained 350 U of *E. coli* DNA ligase (TAKARA BIO). The intra-molecular ligation was performed at 16°C for 2 h. The ligated DNA was precipitated with ethanol, dissolved in 10 μl of distilled water, and used to transform *E. coli* DH5-α cells.

### 2.7. Antibiotic selection

Transformed *E. coli* were selected on LB plates that contained chloramphenicol (Cm), and the QIAprep Spin Miniprep Kit (QIAGEN) was used to isolate the plasmid from cultures of transformed cells.

### 2.8. DNA digestions and sequencing

**Figure 3.**
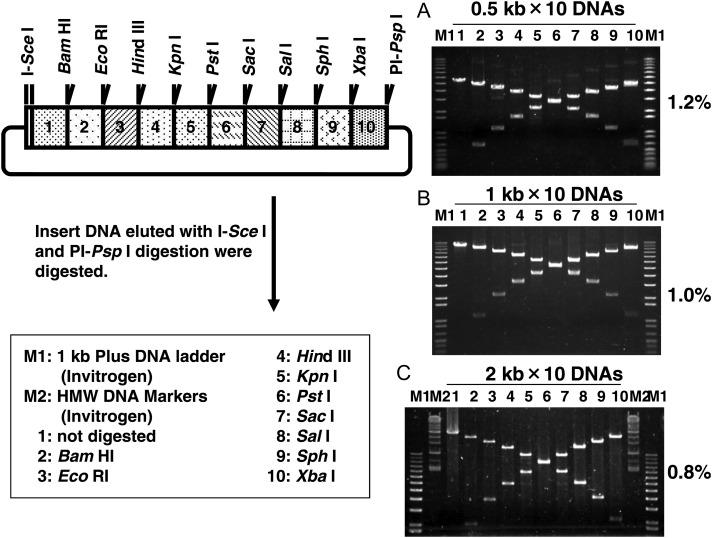
Conformation of the PRESSO products. In each of three experiments, 10 different DNA fragments were ligated in an array along with a plasmid vector; the resulting plasmids were used to transform *E. coli*. Within each experiment; the lengths of all 10 DNA fragments were very similar 0.5, 1, or 2 kb; the inserts on resultant plasmids were 5, 10, or 20 kb, respectively, and were purified after the I-*Sce*I and PI-*Psp*I digestion; the isolated inserts were then used in 10 separate restriction digestion; each enzyme indicated was used to cut at 1 of the 10 segments in each of the three inserts. These restriction sites were designed in the primers for donor DNA amplification. The digested patterns were represented for the sequential ligation of 10 (A) 0.5, (B) 1, or (C) 2 kb DNA fragments. The lengths of digested DNA fragments were consistent with in the lengths of each individual donor DNA molecule listed in Supplementary Table S2. A *Sfi*I site is also located at each connection between each pair of donor DNAs.

The *Sfi*I and I-*Sce*I digestions have been described above. Many restriction enzymes—*Xba*I, *Bam*HI, *Eco*RI, *Hin*dIII, *Kpn*I, *Pst*I, *Sac*I, *Sal*I, *Sph*I, and PI-*Psp*I (TOYOBO)—were used to confirm structure of the resultant plasmid (Fig. 3). The digestions were carried out according to the manufacturer's protocols. Each resultant plasmid was digested with I-*Sce*I and PI-*Psp*I to separate the insert DNA from the vector DNA. The insert DNA was then purified; aliquots of the purified insert DNA were then digested with each restriction enzyme in separate single-enzyme reactions (Fig. 3). The products of each digestion reaction were resolved via gel electrophoresis. The length of each product was compared with two sets of DNA size markers—1 kb plus DNA ladder (Invitrogen) and HMW DNA marker (Invitrogen)—to confirm the structure of the insert DNA. The size of each genomic DNA fragment that was included in the PRESSO array is listed in Supplementary Table S2. To estimate the sequence accuracy of insert DNA, four resultant plasmids with a 20-kb insert consisting of the array of 2.0 kb segments were sequenced. DNA sequences were determined using a Model 3730*xI* DNA analyzer (Applied Biosystems, Forster City, CA, USA). Sequencing reactions were performed with the BigDye® Terminator version 3.1 Cycle Sequencing Kit (Applied Biosystems) according to the manufacturer's instructions.

## 3. Results

### 3.1. Experimental design and overview of a sequential ligation of multiple DNA fragments

We developed a PRESSO method that was based on non-palindromic sticky ends. In summary, there are three steps in PRESSO (Fig. 1). First, a biotin-conjugated linker DNA with a meganuclease restriction site, e.g. the I-*Sce*I site, and a non-palindromic end cleaved by a restriction enzyme, e.g. *Sfi*I or *Bst*XI, is attached to streptavidin-linked beads. Secondly, each DNA fragment digested with *Sfi*I is then ligated, in an ordered sequence of iterative ligation reactions, to the growing end of the DNA construct, which is attached to the streptavidin-linked beads via a biotin label at the opposite end. Thirdly, after the vector sequences are added in the final sequential ligation step, the biotin-conjugated spacer DNA is removed from the construct via I-*Sce*I-catalyzed digestion, and the full-length linear DNA construct is circularized via an intra-molecular ligation to form a circular plasmid that is used to transform *E. coli*. Below, we describe the PRESSO method in detail.

Non-palindromic sequences recognized by some restriction enzymes, such as *Sfi*I and *Bst*XI, are used in PRESSO. Cleavage at such sites can generate any desire 3′-overhanging single-stranded sequences; these overhangs are derived from the bp in the middle of the recognition site. For instance, the restriction enzymes, *Sfi*I and *Bst*XI, recognize the sequences GGCCNNNNNGGCC and CCANNNNNNTGG, respectively, and produce 3′-overhanging ends comprising bases, which are denoted ‘N’, from the middle bp; any nucleotide can occupy any ‘N’ position in an *Sfi*I or *Bst*XI site, and these enzymes produced three or four ‘N’ base-overhang ends, respectively. Non-complementary overhanging bases at each end of each DNA molecule prevent an intra-molecular ligation. In contrast, if the overhanging end of the donor molecule is complementary to the overhanging end of the acceptor molecule, the unpaired bases can form Watson–Crick base pairs between the donor and acceptor DNA molecules, and iterative ligation of such donor–acceptor pairs can produce an array of many DNA fragments that are arranged in defined orientations within a defined array sequence.

PRESSO is carried out on a solid-phase, metallic streptavidin-conjugated beads, because these beads facilitate the removal of reaction intermediates after each ligation reaction, and this process precludes intra-molecular ligation (Fig. 1). The solid phase permits repeated cycles of digestion, purification, and ligation. Streptavidin-conjugated magnetic beads were chosen as the solid phase, because streptavidin binds with high affinity to biotinylated oligonucleotides, and the interaction is sufficiently strong and stable during DNA manipulations.^14^ The beads were also convenient for experimental handling, because they do not bind unlabelled DNA molecules. The initial double-strand DNA molecule (acceptor DNA) was designed to contain a biotinylated 5′-end and 3′-overhanging bases. The acceptor DNA was amplified with a 5′-biotinylated oligonucleotide and unlabelled primers, was cleaved with a non-palindromic restriction enzyme to generate a 3′-overhanging sequence that was complementary to the overhanging sequence on the first donor DNA molecules, and was then attached to the streptavidin beads.

The donor DNA can be prepared either by subcloning into a vector that contains non-palindromic restriction sites or by PCR amplification with appropriate primers. Subsequent digestion of the vector or PCR products with the non-palindromic enzyme, such as *Sfi*I, generates the donor DNA molecules with sticky ends. We purified donor DNA fragments by electrophoresis to remove undigested donor molecules and to remove any cleaved vector fragments that have overhang ends complementary to those of the donor molecules. These purification steps are especially critical for high yields when attempting to join multiple DNA fragments in a defined sequence, because ‘terminated’ molecules will accumulate as a result of incomplete digestion in each ligation cycle, and these ‘terminated’ molecules reduce the yield of the desired molecule. To avoid this problem, we used 5′-biotinylated primers to PCR amplify the donor molecules (Fig. 2); the PCR products were digested with *Sfi*I, then mixed with streptavidin-linked beads, and finally, the unbound fully digested donor fragments were recovered from the supernatant; in contrast, ‘terminated’ partially digested molecules remained bound to the beads. This procedure allowed for the selection of completely digested non-palindromic ends and the successful sequential joining of 10 donor DNA molecules. The purified donor DNA was ligated to the 3′-end of acceptor DNA, which was bound to the beads. After each ligation, the liquid solution was removed and the beads were washed with buffer to accommodate addition of the next donor DNA molecules with non-palindromic ends. Repeated cycles of this procedure allowed the sequential addition of DNA fragments onto the growing acceptor molecules, which were fixed to the solid phase.

The release of the final composite DNA molecules from the solid support was achieved via digestion with the meganuclease I-*Sce*I, and an I-*Sce*I site had been engineered into the initial biotin-labelled linker acceptor fragment and into the final donor vector fragment. The I-*Sce*I restriction enzyme recognizes an 18-bp sequence, which is rarely present in any naturally occurring genome. Other commercially available meganucleases, such as I-*Ceu*I, could also be used for this purpose. Thus, we performed the sequential ligation on the solid phase. Here, as an example, we connected 10 DNA fragments derived from Chromosome 5 of the Arabidopsis genome.

### 3.2. Construction of a plasmid carrying 10 DNA fragments from Arabidopsis

We conducted three prototypical experiments; for each experiment, 10 different donor DNA fragments all of approximately the same length (0.5, 1.0, or 2.0 kb in Experiments A, B, and C, respectively; Fig. 3) were prepared, and each contained a unique restriction site so that the structure of each final plasmid construct could be determined (Supplementary Table S2). On Day 1, we amplified the biotin-conjugated spacer DNA, which included I-*Sce*I and *Sfi*I recognition sequences from the pUC19-I-*Sce*I plasmid and was used for all three experiments; this DNA was digested with *Sfi*I and attached to streptavidin-coated beads as the initial acceptor DNA. For each experiment, 10 unique fragments were prepared as donor DNA fragments; within an individual experiment, all 10 fragments were approximately the same length (0.5, 1.0, or 2.0 kb), but each had a distinct *Sfi*I site and a unique restriction site engineered into the primer. For all experiments, all amplified donor DNA fragments were digested with *Sfi*I, to produce distinct 3′-overhanging bases. Because undigested DNA fragments were removed from the solution by the streptavidin-linked beads, digested fragment could be purified from the supernatants and used as donor DNA molecules. On Day 2, five of each set of 10 DNA fragments were sequentially ligated to acceptor DNA on the streptavidin-linked beads; on Day 3, the remaining five donor molecules from each set of 10 were ligated to the acceptor arrays. After each sequential ligation reaction, the beads were washed, and the newly elongated construct was used as the acceptor molecule in the next ligation. The iterative reactions resulted in three separate arrays, each with 10 distinct DNA segments aligned in defined orientations and defined sequences. In each of the three experiments, the modified pHSG299 vector (pHSG299_CSPS), which encoded a chloramphenicol resistance gene and I-*Ceu*I, I-*Sce*I, PI-*Psp*I, and PI-*Sce*I recognition sequences, was used as the last donor molecule in the final sequential ligation.^15^ The vector was digested with *Sfi*I and ligated to the 3′-overhang bases on final segment of the insert array, which was still attached to the streptavidin-linked beads. Each of the three final products of the sequential ligations was cleaved with I-*Sce*I to release it from the original biotin-labelled acceptor molecule and therefore, the streptavidin-linked beads. These free linear molecules, which each contained 10 distinct segments and a vector backbone, were circularized via an intra-molecular ligation; the circular constructs were then used to transform *E. coli*. The *E. coli* cells were cultured on LB plates that contained chloramphenicol overnight. On Day 4, chloramphenicol-resistant clones were isolated for plasmid preparation.

### 3.3. Confirmation of plasmids carrying 10 DNA fragments

To determine whether the plasmids contain our expected sequences, we digested the plasmid with restriction enzymes specific for the 10 segments within each array. First, we separated the insert DNA, the array of 10 segments, from the pHSG299_CSPS backbone by digestion with I-*Sce*I and PI-*Psp*I, which was followed by elution via gel electrophoresis. To determine whether arrays were arranged as expected, we performed 10 separate digests for each of the three predicted inserts; each digest contained a distinct restriction enzyme that was targeted to a specific segment within an array (Fig. 3). The predicted sizes of the DNA segment within the arrays containing 0.5, 1, or 2 kb segments are listed in Supplementary Table S2. The sizes of the DNA fragments that resulted from the restriction digests were consistent with these estimated segment lengths; therefore, each of the three inserts seemed to include the expected 10 segments in the expected order and orientation (Fig. 3). As we were also anxious about the efficiency of the sequential ligation, we investigated how many plasmids contained all 10 DNA of the expected segments in a single experiment. To assess this, we isolated 12 plasmids carrying the inserts with an array of 2.0 kb segments, and the plasmids were digested with two restriction enzymes, I-*Sce*I and PI-*Psp*I, to separate the insert from the vector backbone. For arrays generated from the 2-kb fragments, the insert DNA from 9 of the 12 plasmids was ∼19.8 kb length, the insert DNA from one plasmid was ∼2 kb length, and other two plasmids carried no insert, suggesting that ∼75% of these transformants contained the expected constructs.

**Figure 4.**
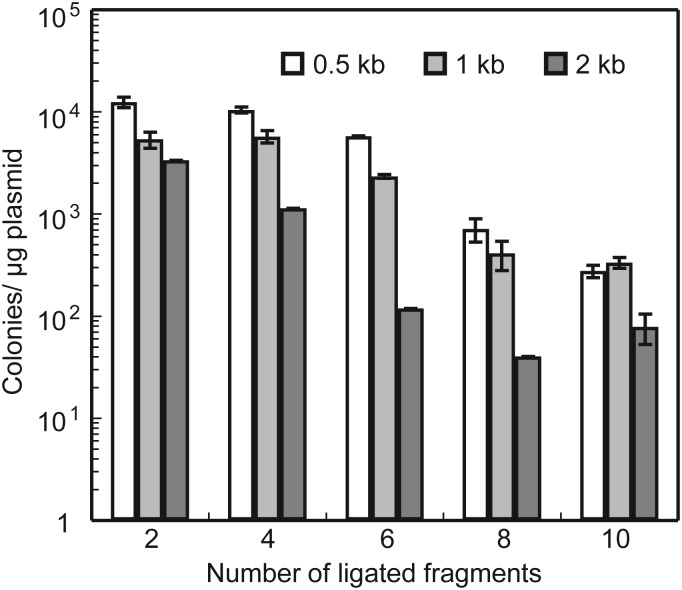
Effects of DNA length and ligation cycles. We ligated 2–10 DNA fragments of 0.5, 1.0, or 2.0 kb segments into the vector pHSG299_CSPS, and then transformed *E. coli*. The number of transformed colonies was shown. The error bars are standard deviations (SD).

To estimate transformation efficiency of the plasmids obtained using the PRESSO method, we connected 2–10 DNA fragments to produce plasmids harbouring the inserts with an array of 0.5, 1.0, or 2.0 kb segments. In any case of PRESSO with <10 segments with up to 2.0 kb inserts, we found more than tens of colonies, even though increasing size of DNA fragments and cycling reactions reduced the efficiency (Fig. 4). Further, we isolated plasmids and investigated the insert length as described above. When we connected more than six DNA fragments using the PRESSO reactions, some plasmids included unexpected inserts. However, even when we connected 2 kb DNA fragments into an array of 10 segments, at least 10 colonies carrying our desired plasmid with correct size were isolated in our study. Thus, the efficiency of PRESSO is sufficient enough to connect multiple DNA fragments.

Further, to investigate the accuracy of the PCR and the ligation reactions, we pick up 4 of 9 correct plasmids as examples, and determined the sequence of inserts within the arrays containing 2 kb segments. One of the four plasmids carried the insert with a correct sequence, and other three plasmids included a couple of errors in the insert sequences. We found total of five errors only in the junction sequences, which were originated as synthetic oligonucleotide PCR primers to amplify multiple DNA fragments. The errors found in the nucleotide sequence produced by the chemical synthesis are reportedly between 160 and 1394 bp per error.^10^ In contrast, PRESSO resulted in a ∼15 840 correct bp for each incorrect bp based on the DNA sequence data; as total of five errors were detected in four clones of 19.8 kb inserts, and the error ratio was estimated as five errors/4 × 19.8 kb = 1/15 840. However, the errors in junction sequences ordinary will not affect the further analyses.

## 4. Discussion

Here, we describe PRESSO, a method for arranging multiple DNA fragments into a single-defined array; this method is an up-to-date technology for handling multiple genes. The solid phase allows researchers to conduct iterative sets of reactions; each set includes a restriction digest, purification, and ligation; in this way, multiple fragments can be arranged into a single-defined array. In this study, we produced a large plasmid that contained a 5, 10, or 20 kb insert, which comprised 10 segments in defined orientations and a defined order. Ligation reactions that join sets of multiple DNA fragments are carried out as a routine task in our laboratory (data not shown). By combining PRESSO with standard cloning techniques, we were able to ligate 22 DNA fragments together in an array that encoded seven functional genes involved in astaxanthin synthesis, and confirmed that this construct indeed functions in transgenic plants^15^ (also see below).

PRESSO has some advantages over other methods of DNA cloning; these advantages include high accuracy and efficiency. Although many methods for ligating multiple DNA fragments together have been developed previously, some of these methods that involve non-palindromic enzymes or Gateway systems can be used to connect only three or four DNA fragments together.^16,17^ These step-by-step methods are more time consuming than the PRESSO method. In methods, such as PAM or the In-Fusion system, all reactions were performed in a single tube without a solid phase;^10–12^ consequently, all DNA fragments are ligated together simultaneously, and the methods are faster than PRESSO. However, these methods that lack stepwise reactions produce undesired DNA sequences, caused by disorder or mismatched ligations. In fact, with the In-Fusion system, the number of transformants recovered decreases as the number of DNA fragments increases.^11^ In contrast, PRESSO, which depends on stepwise reactions on a solid phase, allows us to connect up to 10 cassettes precisely as shown in this study, and a longer DNA fragment in a plasmid can be prepared by ligating building blocks in which up to 10 DNA fragments are incorporated using the PRESSO protocol as shown in our previous research.^15^ By using each DNA fragments with distinct overhang ends, we could avoid disorder or mismatched ligations as seen in the previous methods.^10–12^ In our preliminary experiments, although we carried out the ligation reactions with same overhang sequences repeatedly in one assemble, we could not isolate our designed plasmids harbouring multiple DNA fragments; by skipping internal DNA fragments, many plasmids included less DNA segments than those that we desired. Using the distinct overhang ends is essential for the reaction. Recent reports about a fast ligation based on an automatable solid-phase high-throughput (FLASH) system for TALEffector nucleases (TALENs), which encode sequence-specific artificial nucleases, have also allowed for iterative ligations on a solid phase.^18^ Thus, PRESSO is effective for repeated ligation reactions.

The PRESSO allows us to connect multiple DNA fragments together with the high sequence accuracy. In many previous reports, chemical nucleotide syntheses coupled with PCR and ligation reactions have been used to produce giant DNAs.^13,19^ The nucleotide sequences produced by chemical syntheses contain more errors than standard PCR or cloning as described in our results and in a previous report.^10^ Such undesired mutations in the oligonucleotides are also seen in long-range PCR primers, which are synthesized for the polymerase cycling assembly or for production of the overhanging ends. As long-range PCR for overlap extensions in PAM requires a low-fidelity but high-efficiency polymerase, the amplification of >20 kb of DNA often contains multiple errors.^12,20^ In contrast to the previous reports,^12,20^ the PRESSO system can suppress nucleotide substitutions as shown in our results, because DNA fragments can be designed to be short enough to be amplified with a high-fidelity polymerase.

An important goal in biotechnology is to transfer multiple genes into an organism, so that they function *in vivo*. Using PRESSO, we were able to link 24 DNA fragments to create a single construct comprising a plasmid vector, a single enhancer, and seven functional genes involved in astaxanthin synthesis each within a separate cassette that included a promoter and a operator.^15^ We then introduced the constructed plasmid into plants and confirmed that astaxanthin was synthesized in the transgenic plants.^15^ A decade ago, simultaneous introduction via co-bombardment of multiple independent DNA molecules (genes) into rice cells was described.^21^ However, this co-bombardment approach required that many transformant populations had to be generated and screened to recover the desired transgenic plants for the presence of multiple genes, because the transgenic plants that harboured the desired combination of genes were very rare. This type of strategy is feasible for only some crops, such as rice or tobacco plants, that are readily transformed with high efficiency. In contrast, the PRESSO system described here will facilitate the introduction of a set of genes for a certain metabolic pathway from one species into another species even when the host species supports only low transformation rates. This capability system will open up new opportunities for the production of valuable materials in an easily manageable organism for both basic research and industrial purposes.

A RIKEN Arabidopsis full-length (RAFL) cDNA library (RIKEN, Japan) is a well-established library generated from the Arabidopsis genome; this library covered ∼17 000 of the protein-coding sequences from Arabidopsis, and each sequence was constructed with an *Sfi*I site.^22,23^ Such full-length cDNA clones engineered with a non-palindromic restriction enzyme site can be used for PRESSO without PCR amplification of genes. By using available DNA clones, PRESSO becomes a powerful tool because it is quicker, more precise, and more cost-effective than the previously described methods.

To generate transgenic organisms that express multiple heterologous genes, adaptor cassettes that contain a promoter or reporter may be required. By coupling PRESSO with such cassettes that also include sequences complementally to the ends of cDNA clones, many cDNAs could be directly ligated together in tandem. In our PRESSO system described here, each donor DNA molecule contains designed linker sequences. We believe that this strategy is not problematic in most of the cases, especially for the joining functional genes (as demonstrated in the previous reports^15^). However, if a specific junction sequence is required, as in the case of in-frame ligation of certain protein-coding sequences, Type IIS restriction endonucleases, such as FokI, could be used. Such nucleases cut DNA at the outside sequence of their non-palindromic recognition sites. Site-directed mutagenesis is another approach that could be used to change a sequence as desired after a based plasmid has been constructed.

Multiple PRESSO reactions can be carried out in parallel. Currently, we are working on the simultaneous syntheses of multiple sequential ligation sets; these sets were designed based on network analyses, such as transcriptome or metabolome studies.^3,4,6,7^ Our approach will greatly facilitate the comprehensive characterization of gene function that is currently underway in various organisms.

## Supplementary Data

Supplementary Data are available at www.dnaresearch.oxfordjournals.org.

## Funding

This work was supported by the ‘Innovation Technology for the Earth Program, Development of Transgenic Plants for Production of Industrial Materials Project’ from the New Energy and Industrial Technology Development Organization (NEDO) as a part of the ‘Green Biotechnology Program’, in coordination with the Ministry of Economy, Trade, and Industry (METI); it was also supported in part by the Kazusa DNA Research Institute Foundation.

## Supplementary Material

Supplementary Data
