# Precise Sequential DNA Ligation on A Solid Substrate: Solid-Based Rapid Sequential Ligation of Multiple DNA Molecules

**DOI:** 10.1093/dnares/dsu037

**Published:** 2014-11-16

**Authors:** Eiji Takita, Katsunori Kohda, Hajime Tomatsu, Shigeru Hanano, Kanami Moriya, Tsutomu Hosouchi, Nozomu Sakurai, Hideyuki Suzuki, Atsuhiko Shinmyo, Daisuke Shibata

DNA RESEARCH **20**, 583–592, (2013); doi:10.1093/dnares/dst032

There was an error in the Materials and methods in section 2.7 on page 586. The antibiotic should be kanamycin not chloramphenicol and the correct text is: Transformed *E. coli* were selected on LB plates that contained kanamycin (Km), and the QIAprep Spin Miniprep Kit (QIAGEN) was used to isolate the plasmid from cultures of transformed cells.

