# Supplementary material for: Precise Sequential DNA Ligation on A Solid Substrate: Solid-Based Rapid Sequential Ligation of Multiple DNA Molecules
Source: DNA Res. 2013 Jul 29;20(6):583–92. doi: 10.1093/dnares/dst032 (PMC3859325; doi:10.1093/dnares/dst032)
Supplement: Supplementary Data [file supp_20_6_583__index.html]

Precise Sequential DNA Ligation on A Solid Substrate: Solid-Based Rapid Sequential Ligation of Multiple DNA Molecules — Supplementary Data 

# Precise Sequential DNA Ligation on A Solid Substrate: Solid-Based Rapid Sequential Ligation of Multiple DNA Molecules

## Supplementary Data

Supplementary Data

**Files in this Data Supplement:**

- Supplementary Table 1 - pdf file
- Supplementary Table 2 - pdf file
