# Supplementary material for: Precise Sequential DNA Ligation on A Solid Substrate: Solid-Based Rapid Sequential Ligation of Multiple DNA Molecules
Source: DNA Res. 2013 Jul 29;20(6):583–92. doi: 10.1093/dnares/dst032 (PMC3859325; doi:10.1093/dnares/dst032)
Supplement: Supplementary Data [file supp_dst032_dst032supp_table1.pdf]

Supplemental Table 1. Specific oligonucleotide primers used for the sequential ligations, PCR, and cloning.

primer name	primer sequence	restriction enzyme	
I-SceI-F	AATTCTAGGGATAACAGGGTAATCTGCA		for I-SceI site
I-SceI-R	GATTACCCTGTTAGCCCTAG		for I-SceI site
PUC-N-u	GAGTGGACGATTGGCAGAAGCAGCTGGCACGACAGGTTT		for initial acceptor
PUC-CS	AAGGAAAAAAGGCCACCCAGGCCTGTAAAACGACGGCCAGT	I-*Sce*I	for initial acceptor
CSPS-N2-CAA-u	GAGTGGACGATTGGCAGAAGGCCACAAAGGCCTGGCAAACAGCTATTATGGGTATTATGG	PI-*Psp* I	for last donor (vector)
CSPS-C2-u	GAGTGGACGATTGGCAGAAGTAGGGATAACAGGGTAATTCTTCTTCG		for last donor (vector)
2k			
I F2k-GGG-u	GAGTGGACGATTGGCAGAAGGCCAGGGAGGCCATGGACTAAGAGACAGCTTGGC		
I R-CTT-u	GAGTGGACGATTGGCAGAAGGCCAAAGAGGCCGGTGTTCAGATCCACTAAAGGC		
II F2k-CTT-u	GAGTGGACGATTGGCAGAAGGCCACTTAGGCCGGATCCCTATTGAAACCGGACCAACG	*BamH I*	
II R-ACA-u	GAGTGGACGATTGGCAGAAGGCCATGTAGGCCGGGAAATGTGGGGCTAAGAT		
III F2k-ACA-u	GAGTGGACGATTGGCAGAAGGCCAACAAGGCCGAATTCCCGACGACTCAAAATCTTATCC	*EcoR I*	
III R-TCG-u	GAGTGGACGATTGGCAGAAGGCCACGAAGGCCATCTGGACAGAGGAGTTCAGGT		
IV F2k-TCG-u	GAGTGGACGATTGGCAGAAGGCCATCGAGGCCAAGCTTGGGTACTATTACGGAATACGGG	*Hind III*	
IV R-AAC-u	GAGTGGACGATTGGCAGAAGGCCAGTTAGGCCCTACAGCCATCTTACAAAGCCC		
V F2k-AAC-u	GAGTGGACGATTGGCAGAAGGCCAAACAGGCCGGTACCCGCCATTGACAGTAGTCGTAAC	*Kpn I*	
V R-GGA-u	GAGTGGACGATTGGCAGAAGGCCATCCAGGCCGAGGTGGTTGGTTGTACCGTAT		
VI F2k-GGA-u	GAGTGGACGATTGGCAGAAGGCCAGGAAGGCCCTGCAGAGCTTCAAGATCAAGTGAAGCCA	*Pst I*	
VI R-GCC-u	GAGTGGACGATTGGCAGAAGGCCAGGCAGGCCCGGCACAATCTTACAATGATG		
VII F2k-GCC-u	GAGTGGACGATTGGCAGAAGGCCAGCCAGGCCGAGCTCCCCATCACAATAACCTCTTGGC	*Sac I*	
VII R-CAC-u	GAGTGGACGATTGGCAGAAGGCCAGTGAGGCCGTCTAGGCACAGGCTTGAAAAG		
VIII F2k-CAC-u	GAGTGGACGATTGGCAGAAGGCCACACAGGCCGTCGACTGCGAAAATGTATACACACTAATCT	*Sal I*	
VIII R-TTC-u	GAGTGGACGATTGGCAGAAGGCCAGAAAGGCCTTTCTTGTTATATATGGTTAGCCG		
IX F2k-TTC-u	GAGTGGACGATTGGCAGAAGGCCATTCAGGCCGCATGCCTGGTCATGTGTGCTTTCGT	*Sph I*	
IX R-AGG-u	GAGTGGACGATTGGCAGAAGGCCACCTAGGCCCCACACATGTCGGATCTGTT		
X F2k-AGG-u	GAGTGGACGATTGGCAGAAGGCCAAGGAGGCCTCTAGAGTGTTGATTCTTGACCAGCTCC	*Xba I*	
X R-CAA-u	GAGTGGACGATTGGCAGAAGGCCATTGAGGCCGACAAGTGTAGAACGAAGGGGT		
1k F			
I F1k-GGG-u	GAGTGGACGATTGGCAGAAGGCCAGGGAGGCCTCTGATCACGAATCAGATCCA		
II F1k-CTT-u	GAGTGGACGATTGGCAGAAGGCCACTTAGGCCGGATCCCGTAGGAGAAGAAAAGGAAACG	*BamH I*	
III F1k-ACA-u	GAGTGGACGATTGGCAGAAGGCCAACAAGGCCGAATTCTGGCTTTCTTCCTGATGGTC	*EcoR I*	
IV F1k-TCG-u	GAGTGGACGATTGGCAGAAGGCCATCGAGGCCAAGCTTCATCACCAATTTATGTTCCTTTC	*Hind III*	
V F1k-AAC-u	GAGTGGACGATTGGCAGAAGGCCAAACAGGCCGGTACCGTACGATTTTGGGTCCAATCAC	*Kpn I*	
VI F1k-GGA-u	GAGTGGACGATTGGCAGAAGGCCAGGAAGGCCCTGCAGGCGCGTTCATATATCGTTGC	*Pst I*	
VII F1k-GCC-u	GAGTGGACGATTGGCAGAAGGCCAGCCAGGCCGAGCTCTCACCTCAAATTGTGCGAAT	*Sac I*	
VIII F1k-CAC-u	GAGTGGACGATTGGCAGAAGGCCACACAGGCCGTCGACGGACTTATTTACAACCCAATGCC	*Sal I*	
IX F1k-TTC-u	GAGTGGACGATTGGCAGAAGGCCATTCAGGCCGCATGCTTGTTGGTGAGCACTTTGTAGA	*Sph I*	
X F1k-AGG-u	GAGTGGACGATTGGCAGAAGGCCAAGGAGGCCTCTAGAAGGAAGTGGTGTGGGATTCT	*Xba I*	
0.5k			
I F0.5k-GGG-u	GAGTGGACGATTGGCAGAAGGCCAGGGAGGCCCCATACGCTAAGGCTTGATGTC		
II F0.5k-CTT-u	GAGTGGACGATTGGCAGAAGGCCACTTAGGCCGGATCCCTCTGGTTAATCATTCATCATGC	*BamH I*	
III F0.5k-ACA-u	GAGTGGACGATTGGCAGAAGGCCAACAAGGCCGAATTCGCTTCTGTGGATTTCTTTTCCG	*EcoR I*	
IV F0.5k-TCG-u	GAGTGGACGATTGGCAGAAGGCCATCGAGGCCAAGCTTCAACAGAATCAGTCCAACACAG	*Hind III*	
V F0.5k-AAC-u	GAGTGGACGATTGGCAGAAGGCCAAACAGGCCGGTACCCCTGGAATCTGTTATGTACCACAC	*Kpn I*	
VI F0.5k-GGA-u	GAGTGGACGATTGGCAGAAGGCCAGGAAGGCCCTGCAGGCATTTTCTACGCTAAAGCAGTC	*Pst I*	
VII F0.5k-GCC-u	GAGTGGACGATTGGCAGAAGGCCAGCCAGGCCGAGCTCATGGAGTCCTGAAGAAGACGAG	*Sac I*	
VIII F0.5k-CAC-u	GAGTGGACGATTGGCAGAAGGCCACACAGGCCGTCGACTTCGTCATATTTTGTGATTCG	*Sal I*	
IX F0.5k-TTC-u	GAGTGGACGATTGGCAGAAGGCCATTCAGGCCGCATGCCTGGATATAAGTTCCGGCGA	*Sph I*	
X F0.5k-AGG-u	GAGTGGACGATTGGCAGAAGGCCAAGGAGGCCTCTAGATGACAGAAAATGAGTACAAAAGTG	*Xba I*	
Unv1-biotin	biotin-GAGTGGACGATTGGCAGAAG		

Though the VIII F1k-CAC-u and VIII F0.5k-CAC-u have some complementary sequences on the Arabidopsis genome, a single product was amplified from the K8K14 TAC clone. Red triplet sequences indicate "NNN" on *Sfi*I sites. Blue colored sequences are corresponded to those on the Arabidopsis genome.
