# Supplementary material for: Precise Sequential DNA Ligation on A Solid Substrate: Solid-Based Rapid Sequential Ligation of Multiple DNA Molecules
Source: DNA Res. 2013 Jul 29;20(6):583–92. doi: 10.1093/dnares/dst032 (PMC3859325; doi:10.1093/dnares/dst032)
Supplement: Supplementary Data [file supp_dst032_dst032supp_table2.pdf]

Supplemental Table 2. Information on the 0.5-kb, 1.0-kb, and 2.0-kb DNA fragments used as donor molecules in the sequential ligations

PCR product	FW primer	RV primer	template BAC clone	length (bp)	chr	position	position	restriction enzyme
2.0 kb								
linker (I-*Sce* I, GGG)	PUC-N-u	PUC-CS	pUC19 I-*Sce*I	242				I-*Sce*I
I (GGG-CTT)	I F2k-GGG-u	I R-CTT-u	K21H1	1960	5	26763526	26765485	*BamH I*
II (CTT-ACA)	II F2k-CTT-u	II R-ACA-u	K21H1	1951	5	26803750	26805700	*EcoR I*
III (ACA-TCG)	III F2k-ACA-u	III R-TCG-u	K21H1	1955	5	26808439	26810394	*Hind III*
IV (TCG-AAC)	IV F2k-TCG-u	IV R-AAC-u	K21H1	2022	5	26811047	26813068	*Kpn I*
V (AAC-GGA)	V F2k-AAC-u	V R-GGA-u	K21H1	2042	5	26816362	26818403	*Pst I*
VI (GGA-GCC)	VI F2k-GGA-u	VI R-GCC-u	K8A10	1744	5	26745022	26746765	*Sac I*
VII (GCC-CAC)	VII F2k-GCC-u	VII R-CAC-u	K8K14m2	2034	5	26852589	26854622	*Sal I*
VIII (CAC-TTC)	VIII F2k-CAC-u	VIII R-TTC-u	K8K14m2	2000	5	26891605	26893604	*Sph I*
IX (TTC-AGG)	IX F2k-TTC-u	IX R-AGG-u	K8K14m2	1963	5	26899776	26901738	*Xba I*
X (AGG-CAA)	X F2k-AGG-u	X R-CAA-u	K9I9	2028	5	26923137	26925165	PI-*Psp* I
vector (CAA, PI-*Psp* I, I-*Sce* I)	CSPS-N2-CAA-u (PI-*Psp* I)	CSPS-C2-u (I-*Sce* I)	pACYC184 CSPC	2337				
1.0 kb								
I (GGG-CTT)	I F1k-GGG-u	I R-CTT-u	K21H1	1002	5	26764484	26765485	*BamH I*
II (CTT-ACA)	II F1k-CTT-u	II R-ACA-u	K21H1	1028	5	26804673	26805700	*EcoR I*
III (ACA-TCG)	III F1k-ACA-u	III R-TCG-u	K21H1	1003	5	26809392	26810394	*Hind III*
IV (TCG-AAC)	IV F1k-TCG-u	IV R-AAC-u	K21H1	982	5	26812085	26813068	*Kpn I*
V (AAC-GGA)	V F1k-AAC-u	V R-GGA-u	K21H1	975	5	26817429	26818403	*Pst I*
VI (GGA-GCC)	VI F1k-GGA-u	VI R-GCC-u	K8A10	1048	5	26745718	26746765	*Sac I*
VII (GCC-CAC)	VII F1k-GCC-u	VII R-CAC-u	K8K14m2	975	5	26853648	26854622	*Sal I*
VIII (CAC-TTC)	VIII F1k-CAC-u	VIII R-TTC-u	K8K14m2	1022	5	26892583	26893604	*Sph I*
IX (TTC-AGG)	IX F1k-TTC-u	IX R-AGG-u	K8K14m2	973	5	26900766	26901738	*Xba I*
X (AGG-CAA)	X F1k-AGG-u	X R-CAA-u	K9I9	1049	5	26924114	26925165	PI-*Psp* I
0.5 kb								
I (GGG-CTT)	I F0.5k-GGG-u	I R-CTT-u	K21H1	545	5	26764941	26765485	*BamH I*
II (CTT-ACA)	II F0.5k-CTT-u	II R-ACA-u	K21H1	524	5	26805177	26805700	*EcoR I*
III (ACA-TCG)	III F0.5k-ACA-u	III R-TCG-u	K21H1	478	5	26809917	26810394	*Hind III*
IV (TCG-AAC)	IV F0.5k-TCG-u	IV R-AAC-u	K21H1	549	5	26812520	26813068	*Kpn I*
V (AAC-GGA)	V F0.5k-AAC-u	V R-GGA-u	K21H1	522	5	26817882	26818403	*Pst I*
VI (GGA-GCC)	VI F0.5k-GGA-u	VI R-GCC-u	K8A10	500	5	26746266	26746765	*Sac I*
VII (GCC-CAC)	VII F0.5k-GCC-u	VII R-CAC-u	K8K14m2	491	5	26854131	26854622	*Sal I*
VIII (CAC-TTC)	VIII F0.5k-CAC-u	VIII R-TTC-u	K8K14m2	507	5	26893098	26893604	*Sph I*
IX (TTC-AGG)	IX F0.5k-TTC-u	IX R-AGG-u	K8K14m2	486	5	26901253	26901738	*Xba I*
X (AGG-CAA)	X F0.5k-AGG-u	X R-CAA-u	K9I9	543	5	26924621	26925165	PI-*Psp* I
